# Einteilung und Nomenklatur der aktuellen Materialien zur Kompressionstherapie

**DOI:** 10.1007/s00105-023-05108-7

**Published:** 2023-02-08

**Authors:** K. Protz, S. Eder, S. Läuchli, H. Partsch, M. Stücker, J. Traber, J. Dissemond

**Affiliations:** 1 CompetenzzentrumVersorgungsforschung in der Dermatologie (CVderm), Institut für Versorgungsforschung in der Dermatologie und bei Pflegeberufen (IVDP), Universitätsklinikum Hamburg-Eppendorf (UKE), Hamburg, Deutschland; 2 Klinik für Gefäßchirurgie und Gefäßmedizin, Schwarzwald-Baar Klinikum, Villingen-Schwenningen, Deutschland; 3 Dermatologisches Zentrum Zürich AG, Zürich, Schweiz; 4 Medizinische Universität Wien, Wien, Österreich; 5 Klinik für Dermatologie, Venerologie und Allergologie, Ruhr-Universität Bochum, Venenzentrum der Dermatologischen und Gefäßchirurgischen Kliniken, Bochum, Deutschland; 6 Venenklinik Bellevue Kreuzlingen (VBK), Kreuzlingen, Schweiz; 7 Klinik und Poliklinik für Dermatologie, Venerologie und Allergologie, Universitätsklinikum Essen, Hufelandstr. 55, 45122 Essen, Deutschland

**Keywords:** Intermittierende pneumatische Kompressionstherapie, Phlebologischer Kompressionsverband, Mehrkomponentensysteme, Medizinische adaptive Kompressionssysteme, Ulkus-Strumpfsysteme, Intermittent pneumatic compression, Phlebological compression bandages, Multicomponent systems, Adjustable compression wraps, Ulcer stocking systems

## Abstract

Die Kompressionstherapie ist seit mehreren Hunderten von Jahren ein wesentlicher Bestandteil der konservativen Therapie bei Menschen mit chronischen Wunden und Ödemen der unteren Extremitäten. Die dann eingeleitete Therapie kann in die Phasen der Entstauung, Erhaltung und Prävention unterteilt werden. Die Auswahl der jeweiligen Kompressionsversorgung orientiert sich u. a. an den Therapiephasen, dem klinischen Stadium und Symptomen, den Bedürfnissen Betroffener und deren körperlichen Fähigkeiten. Für die Kompressionstherapie steht heute eine Vielzahl an unterschiedlichen Materialien und Methoden zur Verfügung. Daher gestaltet es sich zunehmend schwieriger, einen Überblick über diese Behandlungsoptionen zu behalten, zumal die verwendete Nomenklatur der Hersteller oft nicht einheitlich ist. In dieser Übersichtsarbeit werden daher die aktuell im deutschsprachigen Raum verfügbaren Materialien und Methoden zur Kompressionstherapie mit ihren Einsatzmöglichkeiten erläutert. Zudem wird eine einheitliche Nomenklatur vorgeschlagen, auf deren Basis eine sachgerechte Dokumentation und Kommunikation aller an der Versorgung von Menschen mit Kompressionstherapie Beteiligten gewährleistet ist.

## Abstract (en)

Compression therapy has been an essential part of conservative therapy for people with chronic wounds and edema of the lower extremities for hundreds of years. The initiated therapy can be divided into the decongestion phase, maintenance phase, and prevention. The choice of the respective compression materials is based, among other factors, on these phases, the clinical stage and symptoms, the needs of the affected person and their physical abilities. Today, a wide range of different materials and methods are available for compression therapy. Thus, it is increasingly difficult to keep an overview of these treatment options, especially since the nomenclature used by the manufacturers is often inconsistent. Thus, the materials and methods for compression therapy currently available in German-speaking countries and their clinical indications are described in this review article. In addition, a uniform nomenclature is proposed, on the basis of which an appropriate exchange between all those involved in the care of people with compression therapy is guaranteed.

Die Kompressionstherapie ist traditionell ein wesentlicher Bestandteil der konservativen Therapie bei Menschen mit chronischen Wunden und Ödemen der unteren Extremitäten. Neben der chronischen venösen Insuffizienz (CVI) können auch viele weitere interdisziplinär relevante Krankheitsbilder wie Niereninsuffizienz, Proteinmangel oder Adipositas zu Ödemen führen. Die dann eingeleitete Therapie lässt sich bei Ödemen in die Phasen der Entstauung und Erhaltung sowie bei venösen Ulzera in die Phasen, Entstauung, Erhaltung und Prävention unterteilen [6, 21]. Die Auswahl der jeweiligen Kompressionsversorgung orientiert sich dabei u. a. an diesen Therapiephasen, den klinischen Beschwerden, den Bedürfnissen Betroffener und deren körperlichen Fähigkeiten. Für die Kompressionstherapie steht heute eine Vielzahl an Materialien und Methoden zur Verfügung. Es gestaltet sich daher zunehmend schwieriger, einen Überblick über diese Behandlungsoptionen zu behalten, zumal die verwendete Nomenklatur der Hersteller oft nicht einheitlich ist.

Im Folgenden werden daher in diesem Übersichtsartikel die aktuell im deutschsprachigen Raum verfügbaren Materialien und Methoden zur Kompressionstherapie und deren Einsatzmöglichkeiten erläutert. Zudem wird auf der Basis eines Expertenkonsenses eine einheitliche Nomenklatur vorgeschlagen, die einen sachgerechten Austausch aller an der Versorgung von Menschen mit Kompressionstherapie Beteiligten gewährleistet (Tab. 1).

**Table 1:** 

**Binden** *Elastische Kompressionsbinden* – Langzugbinden, Dehnbarkeit > 100 % (meist 140–200 %) – Elastische Mullbinden *Unelastische Kompressionsbinden* – Kurzzugbinden, Dehnbarkeit < 100 % (meist im Bereich > 10 und < 100 %) – Zinkleimbinden, Dehnbarkeit < 10 % *Mehrkomponentensysteme* – Systeme mit 1 Binde – Systeme mit 2 Binden – Systeme mit 3 Binden – Systeme mit 4 Binden *Polstermaterialien* – Wattebinden – Schaumstoffbinden – Pelotten – Polstermanschette – Baumwollschlauchverband
**Medizinische adaptive Kompressionssysteme (MAK)**
**Bestrumpfung** *Ulkus-Strumpfsysteme* *Medizinische Kompressionsstrümpfe (MKS)* – Flachgestrickt – Rundgestrickt – Spezielle MKS für Menschen mit CVI und pAVK und/oder Diabetes mellitus *Medizinische Thromboseprophylaxestrümpfe (MTPS)*
**Sonstige** *Intermittierende pneumatische Kompressionstherapie (IPK)*

*CVI* chronische venöse Insuffizienz, *pAVK* periphere arterielle Verschlusskrankheit

## Phlebologischer Kompressionsverband

Als phlebologischen Kompressionsverband (PKV) bezeichnet man Verbände mit verschiedenen Materialien, die ursprünglich für die Therapie von Menschen mit phlebologischen Krankheitsbildern genutzt wurden. Da sich dieser Begriff mittlerweile in Abgrenzung beispielsweise von lymphologischen Kompressionsverbänden etabliert hat, wird er auch bei Menschen genutzt, bei denen keine phlebologische Erkrankung zugrunde liegt, aber ein entsprechender Kompressionsverband angelegt wird. So werden PKV heute auch bei Menschen beispielsweise mit Vaskulitiden, Vaskulopathien oder Necrobiosis lipoidica angewendet [6].

Der PKV wird in der ersten Phase der Kompressionstherapie, der Entstauungsphase, angewendet. Es lassen sich zwei wesentliche therapeutische Aspekte der Kompression differenzieren, für die entsprechend unterschiedliche Behandlungsoptionen möglich sind. Wenn es um die Verbesserung der venösen Hämodynamik geht, werden für die erwünschte Verkleinerung des Venendurchmessers im Stehen und Gehen bei Menschen hohe Druckwerte von mindestens 40 mmHg benötigt. Für Menschen mit Ulcus cruris venosum (UCV) konnte gezeigt werden, dass eine kräftige Kompressionsversorgung mit einem Ruhedruck zwischen 40 und 60 mmHg die besten Ergebnisse erzielt [16, 21]. Zur Ödemreduktion kann eine ausreichende Wirkung auch schon mit Ruhedruckwerten um 20 mmHg erzielt werden [17]. Für PKV ist die überlegene Wirksamkeit der höheren Druckwerte belegt, nicht aber für medizinische Kompressionsstrümpfe [14, 15].

Ein PKV schließt grundsätzlich Fuß- und Sprunggelenk mit ein und reicht entweder bis zum Fibulaköpfchen oder bis zum proximalen Oberschenkel [22]. Ein sach- und fachgerecht ausgeführter PKV, der von den Menschen akzeptiert und entsprechend getragen wird, sollte spätestens nach vier Wochen zu einer erfolgreichen Entstauung führen [28]. Ein PKV kann als Wechsel- oder als Dauerverband ausgeführt werden. Wechselverbände verbleiben idealerweise über Nacht und sind täglich neu anzulegen. Ein Dauerverband wird über mehrere Tage am Bein belassen [22].

Ein PKV kann mit verschiedenen Kompressionsmaterialien ausgeführt werden. Hierbei werden wieder verwendbare oder lediglich einmalig zu nutzende Materialien eingesetzt. Ein PKV, der aus steifem, unnachgiebigem Material besteht, setzt der Beinmuskulatur ein stabiles Widerlager entgegen. Der Effekt der Wadenmuskelpumpe wird dadurch intensiviert und der venöse Rückfluss verbessert. Das Akronym P‑LA-C‑E (P [Pressure]/LA [Layers]/C [Components]/E [Elasticity]) bezeichnet ein Konzept, das eine wichtige Hilfe für die Beurteilung von PKV ist ([16]; Tab. 2).

**Table 2:** 

*P* (Pressure)	Druck, den der PKV auf die Extremität ausübt
*LA* (Layers)	Überlappung der Materialien, sowohl einzelner Komponenten als auch mehrerer übereinander
*C* (Components)	Art der Materialien, aus denen sich die einzelnen Komponenten zusammensetzen
*E* (Elasticity)	Elastizität, die das Material befähigt, einen Druck bei unbewegter Extremität zu erzeugen

In England gab es bereits 1988 die ersten Kompressionssysteme mit vier Binden. Der hierfür verwendete Begriff „Mehrlagenkompression“ wurde ursprünglich aus dem englischen Sprachgebrauch „multi-layered systems“ übersetzt, was inhaltlich allerdings nicht korrekt ist, da jeder PKV aus mehreren Lagen besteht [15]. Bindentouren überlappen sich mindestens um 50 %, durch den Ferseneinschluss am Fuß zum Teil sogar bis zu 3‑mal. Die Anzahl der Lagen beeinflusst die sog. Stiffness (Steifigkeit), also die Fähigkeit des Kompressionsverbands möglichst starr zu bleiben. Je mehr Lagen übereinander angelegt werden, desto mehr ändern sich die elastischen Eigenschaften der gesamten Bandagierung, somit wird der PKV starrer. Hierbei ist es unerheblich, ob eine Binde sich selbst mehrmals überlappt oder mehrere Binden übereinander appliziert werden [22]. Da sich Binden bei jeder Bandagierung überlappen, gibt es somit keinen „Ein-Lagen-PKV“. Stattdessen sollte von einer Ein- oder Mehrkomponentenkompression gesprochen werden. Ein PKV, der lediglich aus zwei Binden derselben Art besteht, ist somit ein Einkomponentenverband aus mehreren Lagen. Es konnte gezeigt werden, dass ein PKV, der aus mehreren verschiedenen Komponenten besteht, effektiver für die Abheilung eines UCV ist, als wenn lediglich eine Komponente eingesetzt wird ([15, 18]; Tab. 3).

**Table 3:** 

Hautschutz, z. B. Baumwollschlauchverband, Polstermanschette
Unter‑/Aufpolsterung, z. B. Pelotten, Watte‑/Schaumstoffbinden, Polstermanschette
Druckerzeugung, z. B. Kurzzug‑, Langzug‑, Zinkleimbinden, Polstermanschette
Fixierung, z. B. Pflasterstreifen, Fixiervlies, kohäsive Binden
Sonstige Stoffe, z. B. Calamin, Zink, Kupferfasern

## Kompressionsbinden

Kompressionsbinden sind im Wesentlichen längselastisch gefertigt und unterscheiden sich hinsichtlich des Dehnungsvermögens. Hierbei wird zwischen Binden, die ein Dehnungsvermögen von über oder unter 100 % haben, unterschieden. Elastische Binden haben ein maximales Dehnungsvermögen von über 100 %; hierzu gehören Langzugbinden. Unelastische Binden haben eine maximale Dehnung von unter 100 %. Neben den Kurzzugbinden gehören hierzu auch die Zinkleimbinden, die mit unter 10 % über fast kein Dehnungsvermögen verfügen ([22]; Tab. 1). Zudem gibt es Polsterbinden und kohäsive, auf sich selbst haftende Binden sowie kombinierte Sets aus mehreren Materialien, sog. Mehrkomponentensysteme. Die meisten Arten von Kompressionsbinden sind in den Breiten 6, 8, 10 und 12 cm sowie in den Längen 5, 6 und 7 m erhältlich. Welche Größe bei der Erstellung eines PKV zum Einsatz kommt, orientiert sich in erster Linie an anatomischen Aspekten wie Fußgröße, Unterschenkellänge und -umfang.

Die oft synonym genutzte Bezeichnung „Bandagen“ für Kompressionsbinden ist nicht korrekt, da Bandagen Hilfsmittel sind, die beispielsweise Gelenke stabilisieren oder deren Funktion unterstützen sollen. Ein PKV mit Kompressionsbinden kann allerdings als (Kompressions‑)Bandagierung bezeichnet werden.

### Langzugbinden

Der Punkt, bis zu dem eine Binde dehnbar ist, nennt man Dehnungssperre. Bei Langzugbinden liegt diese zwischen 140 bis zu 200 %. Diese Binden erzeugen einen hohen Ruhedruck und in Bewegung einen niedrigen Arbeitsdruck. Somit erwirken Langzugbinden bei Muskelkontraktion nur einen geringen Widerstand und erzielen eine geringe rückflussfördernde Wirkung. Bei Bewegung und Veränderung des Umfangs der Extremitäten passt sich die Langzugbinde allerdings an und kann den Druck über mehrere Tage halten. Aufgrund des Risikos von Druckschäden ist es nicht ratsam, ausschließlich Langzugbinden für einen PKV zu verwenden. Zudem ist ihr Einsatz bei immobilen Menschen kritisch zu sehen, da sie aufgrund des hohen Ruhedrucks bei längeren Ruhephasen starke Einschnürungen erzeugen können und es somit zu einer Erhöhung des Thromboserisikos kommen kann. Allerdings können Langzugbinden in Mehrkomponentensystemen verwendet werden, um ein Rutschen des Verbandes zu verhindern und den Anlagedruck durch den kombinierten Einsatz, insbesondere bei dem Tragen über mehrere Tage, besser zu halten.

### Zinkleimbinden

Zinkleimbinden werden feucht angelegt und härten je nach Materialeigenschaften mehr oder weniger aus. Durch die Verdunstung haben sie anfänglich einen angenehm kühlenden Effekt. Die Dehnungssperre liegt bei unter 10 %. Dadurch erzeugen Zinkleimbinden einen niedrigen Ruhedruck, der beim Anlegen gesteuert werden kann, und in Bewegung einen sehr hohen Arbeitsdruck. Es gibt bi-elastische, d. h. gleichzeitig quer- und längselastische sowie unelastische Zinkleimbinden. Bi-elastische Zinkleimbinden sind einfacher anzulegen und härten nicht vollständig aus. Sie hinterlassen allerdings an der Kleidung und in der Umgebung Materialrückstände. PKV mit Zinkleimbinden bewirken durch ihre geringe Dehnbarkeit zu Beginn eine schnelle Entstauung. Der therapierelevante Druck wird aber nur so lange gewährleistet, wie sich der Umfang der Extremität nicht vermindert. Im Verlauf der Ödemreduktion nimmt somit der Druck entsprechend ab. Zudem schränken PKV mit Zinkleimbinden, insbesondere bei unsachgemäßer Anlage, aufgrund ihrer relativen Unnachgiebigkeit die Arbeit der Venenpumpen im Sprunggelenk ein, da der optimale Abrollvorgang behindert wird. Bei den sog. Kompressionsverbänden nach Fischer werden Zinkleim- und Kurzzugbinden kombiniert mit einem Ruhedruck von 50–70 mmHg angelegt [5]. Die Erstellung eines PKV mit Zinkleimbinden erfordert umfassende Materialkenntnisse und Wissen über sachgerechte Anlagetechniken und Erfahrung in deren Anwendung [6].

### Kurzzugbinden

Da bei Kurzzugbinden das Dehnungsvermögen unter 100 % liegt, werden sie als wenig elastisch oder sogar unelastisch bezeichnet. Das wenig dehnbare Bindenmaterial setzt der Muskelkontraktion in Bewegung einen starken Druck entgegen und erwirkt so einen hohen Arbeitsdruck. Im Gegensatz dazu ist der Druck bei ruhendem Bein niedrig. Kurzzugbinden kommen daher überwiegend bei mobilen Menschen zur Anwendung, bei denen durch Muskelkontraktion v. a. der Wadenmuskulatur ein entsprechender Arbeitsdruck gewährleistet ist.

Mit Kurzzugbinden lassen sich PKV mit einem hohen Ruhedruck anlegen. Allerdings fällt der Druck bereits kurz nach Anlage rasch ab, wenn sich das Bein bewegt. So kommt es bei PKV mit Kurzzugbinden bereits innerhalb von 24 h zu einem erheblichen Druckabfall [9, 10, 19]. Wenn der Druck nachlässt, beginnen die Kurzzugbinden aufeinander zu rutschen, und der PKV verliert seine Form. Die Binden lockern sich ebenfalls, wenn sich das Ödem mindert [22]. Die Kompressionstherapie ist aber nur erfolgreich, wenn ein adäquater Druck über eine angemessene Zeit Wirkung entfalten kann. Daher sind PKV mit Kurzzugbinden als Einkomponentenverband in der Entstauungsphase, insbesondere zu Beginn der Ödemreduktion, als Wechselverband täglich zu erneuern.

Die Erstellung eines PKV mit Kurzzugbinden setzt umfassende Materialkenntnisse und Wissen über sachgerechte Anlagetechniken und Erfahrung in deren Anwendung voraus. Aktuelle Studien zeigen allerdings erhebliche Defizite aufseiten der Versorger in der sachgerechten Anlage von PKV mit Kurzzugbinden auf. So wird oftmals weder zuverlässig ein therapierelevanter Druck erzielt, noch der PKV fachgerecht angelegt [8].

Ideal- und Universalbinden sowie elastische Mullbinden sind nicht für die Erstellung von PKV geeignet. Elastische Mullbinden können allerdings bei Vorfuß- und Zehenödemen für die Entstauung als Kompressionsverband der Zehen genutzt werden.

## Mehrkomponentensysteme

**Figure 1:**
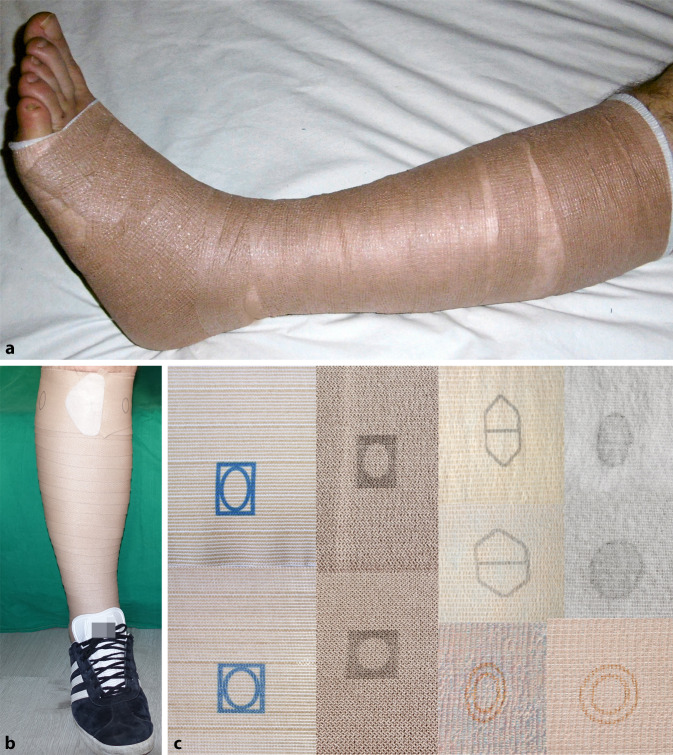

Mehrkomponentensysteme sind seit mehr als zwei Jahrzehnten eine zeitgemäße Alternative zu anderen Kompressionsmaterialien. Mittlerweile sind viele unterschiedliche konfektionierte Sets auf dem deutschsprachigen Markt erhältlich. Diese bestehen aus mehreren Komponenten und beinhalten meist Polster‑, Kompressions- und kohäsive Fixierbinden. Mehrkomponentensysteme kombinieren teilweise Kurz- und Langzugbinden sowie kohäsive Materialien. Solche Systeme bestanden ursprünglich aus vier Binden. Auch wenn die therapeutischen Effekte dieser Systeme oft sehr gut waren, gestaltete sich die Umsetzung in der täglichen Praxis schwierig. Die Kompressionsverbände waren relativ dick, sodass diese oft zu einem Wärmestau sowie Problemen bei der Schuhwahl geführt haben. Im Laufe der Jahre haben sich daher im deutschsprachigen Raum eher Mehrkomponentensysteme mit zwei Binden durchsetzen können (Abb. 1a). Eine neue Entwicklung sind Mehrkomponentensysteme, die lediglich aus einer Binde bestehen und die Eigenschaften von Kurz- und Langzugbinden miteinander kombinieren (Abb. 1b).

Im Gegensatz zu PKV mit Kurzzugbinden erfordert die sachgerechte Erstellung eines effizienten PKV mit Mehrkomponentensystemen keine speziellen Kenntnisse über komplexe Bandagierungstechniken. Zudem ist die Anlage weniger zeitintensiv [20]. Mehrkomponentensysteme sind ursprünglich dafür konzipiert, eine kräftige Kompression von etwa 40 mmHg zu erzeugen und über mehrere Tage zu halten. Einige Hersteller bieten zudem sog. „Lite-Varianten“ an, die bei korrekter Anlage einen Ruhedruck von etwa 20 mmHg erzeugen. Diese Varianten ermöglichen dadurch auch die Versorgung von Menschen mit Risikofaktoren, beispielsweise mit begleitender moderater peripherer arterieller Verschlusskrankheit (pAVK). Eine Kompressionstherapie kann grundsätzlich durchgeführt werden, wenn der Ankle-Brachial-Index (ABI) > 0,5, Knöchelarteriendruck > 60 mmHg oder Zehendruck > 30 mmHg ist. Eine weiterhin sehr wichtige Kontraindikation der Kompressionstherapie ist hingegen die kritische Ischämie (Tab. 4; [2]). Bei Menschen mit pAVK sollte bei dem Einsatz von Kompressionstherapie mit Druckwerten oberhalb 20 mmHg möglichst auch eine Polsterung erfolgen, um die Behandlungssicherheit und den Hautschutz zu erhöhen. Einige der Mehrkomponentensysteme nutzen spezielle Dehnungstechniken oder verfügen über Marker, die eine korrekte Anlage erleichtern sollen (Abb. 1c). Durch diese optische Hilfe kann im Vergleich zu Kurzzugbinden der therapierelevante Anlagedruck einfacher erreicht werden. Aufgrund von kohäsiven oder anderen haftenden Eigenschaften wird der PKV zudem stabilisiert, und es wird einem schnellen Verrutschen vorgebeugt. Ein täglicher Wechsel von Mehrkomponentensystemen ist nicht erforderlich, kostenintensiv und unterbricht zudem den Entstauungsprozess. Je nach Entstauungssituation können solche Systeme bis zu sieben Tage am Unterschenkel verbleiben [6]. Mehrkomponentensysteme sind weder waschbar noch wiederverwendbar. Da sie schneller zu einem Entstauungserfolg führen, kann ihre Anwendung dennoch kosteneffektiver sein, da sich die Therapiezeiten verkürzen und zudem weniger Aufwendungen für Verbandmittel anfallen [4]. Auch für die Praxis der Versorgung haben die Mehrkomponentensysteme viele Vorteile. Die Menschen sind beweglicher im Sprunggelenk, was die Arbeit der Venenpumpen unterstützt, und haben zudem weniger Schuhprobleme, da diese Verbände mit einer oder zwei Binden weniger auftragen als ein unterpolsteter Verband mit Kurzzugbinden [20].

**Table 4:** 

*Fortgeschrittene periphere arterielle Verschlusskrankheit*^a^
*Dekompensierte Herzinsuffizienz*
*Phlegmasia coerulea dolens*
*Septische Phlebitis*

^a^ ABI < 0,5, Knöchelarteriendruck < 60 mmHg, Zehendruck < 30 mmHg, tcpO_2_ < 20 mmHg Fußrücken

*ABI* Ankle-Brachial-Index, *tcpO*_*2*_ transkutane Sauerstoffpartialdruckmessung

## Medizinische adaptive Kompressionssysteme

**Figure 2:**
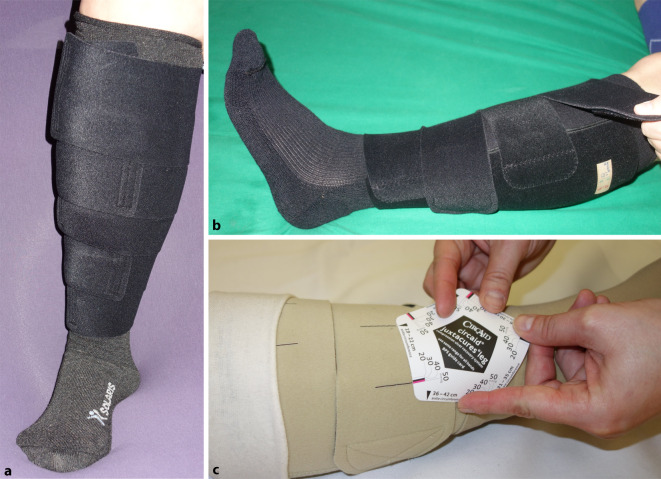

Medizinische adaptive Kompressionssysteme (MAK) wurden bislang auch als Klettbandagen oder Wrap-Verbände bezeichnet. Hier sollte zukünftig für die nachvollziehbare Zuordnung einheitlich die Bezeichnung als MAK genutzt werden. MAK sind eine im deutschsprachigen Raum erst seit einigen Jahren erhältliche weitere Versorgungsoption, die während der Entstauungsphase und je nach Produkt auch in der Erhaltungsphase angewendet werden kann. Es handelt sich um verschiedene Systeme mit Manschetten, die um den Unterschenkel angelegt und mit Klettbändern, -verschlüssen oder Klettpatches verschlossen werden. Bei einigen MAK überlappen sich die Manschettenbänder (Abb. 2a), bei anderen liegen sie parallel zueinander (Abb. 2b). Je nach Hersteller sind MAK mit oder ohne Extra-Fußbandage und (Kompressions‑)Strümpfe bzw. hautschützende Schlauchverbände erhältlich. Produkte ohne zusätzliche Fußbandage haben gegenüber den PKV mit Kompressionsbinden den Vorteil, dass der Betroffene geringere Schuhprobleme hat. Derzeit gibt es MAK für Menschen mit UCV mit einer Druckwertspanne von 20–60 mmHg. Bei einigen MAK ist es möglich, den bestehenden Druck visuell zu überprüfen und gezielt einzustellen (Abb. 2c). Wenn der Beinumfang im Laufe der Therapie durch Ödemreduktion abnimmt, lassen sich die Klettverschlüsse einfach und individuell nachjustieren [6, 13, 20]. Diese adaptive Eigenschaft bedeutet einen Vorteil gegenüber den PKV mit Kompressionsbinden, die bei Druckverlust oder Verrutschen komplett neu anzulegen sind. Menschen, die an ihre Füße gelangen, oder auch Angehörige können diese Adaption nach einer kurzen Edukation oft selbstständig ausführen [3]. Der eigenverantwortliche Umgang mit den MAK kann das Selbstmanagement der Betroffenen steigern und somit deren Adhärenz fördern.

## Polstermaterialien

**Figure 3:**
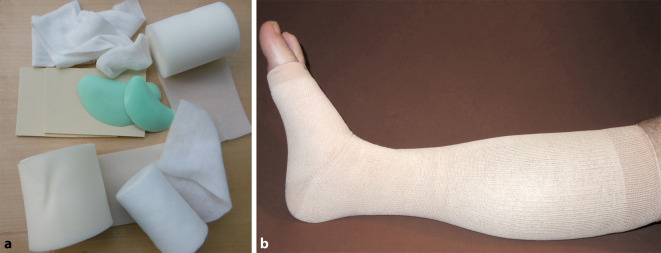

Polstermaterialien können die Haut vor Schnürfurchen und Druckstellen schützen, Unebenheiten oder prominente Vorsprünge ausgleichen und dafür sorgen, dass sich der Druck, den die Kompressionsverbände ausüben, gleichmäßiger verteilt [21]. Daher ist Unter‑, Auf- oder Abpolsterung oft eine wesentliche Komponente der PKV. Unterpolsternde Binden, die oft zusätzliche komprimierende Eigenschaften haben, sind auch integriert in Mehrkomponentensysteme. Bei der Unterpolsterung von PKV kommen Binden aus natürlicher Baumwolle oder synthetischer Watte- sowie Schaumstoffbinden oder sog. Polstermanschetten zum Einsatz (Abb. 3a, b). Während Wattebinden sich gut an die Körperformen anmodellieren lassen, sind Schaumstoffbinden starrer, aber waschbar und somit kosteneffizienter. Diese Polsterbinden sollen einer Hautschädigung vorbeugen und werden in Bereichen mit hohen Druckwerten aufgrund des engen Radius sowie an Arealen eingesetzt, wo die Haut dünner ist und sich prominente knöcherne Strukturen befinden. Hierzu gehören Regionen, an denen der Körper über weniger schützendes Gewebe und Fettgewebe verfügt, beispielsweise der Fußrücken, die Knöchelregion, der Achillessehnenbereich und die Tibiavorderkante. Größere Umfangdifferenzen durch ungleichmäßig geformte Extremitäten sind durch Auf- und Abpolsterung auszugleichen. So trägt ein Ausgleich von Hervorhebungen oder Absenkungen dazu bei, dass der Druck an allen Regionen gleichmäßig einwirkt. Hierfür werden spezielle Druckpolster, sog. Pelotten, eingesetzt. Pelotten können aber auch individuell von entsprechenden Schaumstoffrollen und Platten aus Filz oder Schaumstoff bedarfsgerecht zugeschnitten werden.

Polstermaterialien, die bei einem PKV zum Einsatz kommen, haben somit einerseits die Aufgabe, die Haut gegenüber den Kompressionsbinden abzupolstern. Andererseits sorgen sie für eine Verteilung des Anlagedrucks, was iatrogenen Schädigungen vorbeugt, den PKV effizienter und komfortabler macht [7, 11]. Ähnlich wie Kompressionsbinden können auch Polsterbinden Hautirritationen und Juckreiz durch Reibung auf der Haut auslösen [23]. Der Schutz der Haut vor diesen Effekten kann durch einen Schlauchverband aus Baumwolle und adäquate Hautpflegeprodukte erfolgen, die jeweils direkt auf die Hautoberfläche aufgebracht werden [6]. Die sog. Polstermanschetten verbinden die Eigenschaften der Polsterung und des Hautschutzes.

## Bestrumpfung

Im Gegensatz zu PKV mit Kompressionsbinden liegt der erzielte Druck bei medizinischen Kompressionsstrümpfen (MKS) nicht in der Hand des Anwenders. Diese Produkte gewährleisten nach korrekter Anmessung eine gleichmäßige Druckverteilung in der gewünschten Kompressionsklasse (KKL) (Tab. 5). Im Vergleich zu PKV mit Kompressionsbinden ist die Beweglichkeit im Sprunggelenk weniger eingeschränkt, und Schuhe können besser getragen werden. Vielen Betroffenen ist es zudem möglich, diese Versorgung mit An- und Ausziehhilfen selbstständig oder mit Unterstützung durch die Angehörigen an- und abzulegen. Dadurch werden das Selbstmanagement und die Adhärenz gefördert.

**Table 5:** 

Kompressionsklasse (KKL)	Druck in mmHg	Druck in kPa^a^	Intensität
I	18–21	2,4–2,8	Leicht
II	23–32	3,1–4,3	Mittel
III	34–36	4,5–6,1	Kräftig
IV	49 und größer	6,5 und größer	Sehr kräftig

Quelle: Gütesicherung RAL-GZ 387/1, Januar 2008. Diese Einteilung gilt nur für MKS und nicht für Kompressionsbinden

^a^1 kPa = 7,5 mmHg, 1 mmHg = 0,133 kPa

Der oft verwendete und meist negativ besetzte Begriff „Gummistrümpfe“ ist sachlich nicht korrekt, da in den meisten Strümpfen heute keinerlei Gummibestandteile mehr enthalten sind. Daher sollte dieser Begriff heute nicht mehr verwendet werden [1].

### Strickung

Die heute erhältlichen MKS werden in verschiedenen Strickungen hergestellt. Hier können Rund- und Flachstrick unterschieden werden. Rundgestrickte MKS werden auf einem Strickzylinder mit einer definierten Anzahl an Nadeln ein- und doppelfädig gefertigt. Es können also weder zusätzliche Maschen auf- noch Maschen wieder abgenommen werden. Eine Anpassung an die Beinform ist somit nur begrenzt möglich. Bei außergewöhnlichen Beinumfängen hat diese Fertigungsmethode Grenzen. Im Gegensatz dazu können mit flachgestrickten MKS auch Extremitäten mit sehr kleinen Umfängen und extremen Umfangsänderungen versorgt werden, wie es beispielsweise bei lymphatischen Erkrankungen oft notwendig ist. Beim Flachstrickverfahren wird der MKS an einem Stück flächig gestrickt und abschließend zusammengenäht, sodass im Gegensatz zu den rundgestrickten MKS an der Beinrückseite eine Naht entsteht. Dabei können Maschen auf- oder abgenommen werden. Dies gewährleistet eine Anpassung des Strumpfes an die individuellen Beingegebenheiten. Flachstrickprodukte sind deutlich gröber, fester, dicker und weniger elastisch als rundgestrickte MKS [22]. Eine Neuerung stellt die sog. 3‑D-Stricktechnologie dar. Hiermit können flachgestrickte Varianten auch ohne Nähte produziert werden.

### Ulkus-Strumpfsysteme

**Figure 4:**
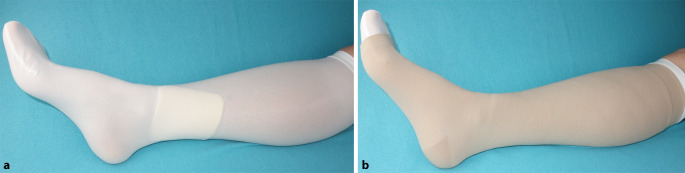

Ulkus-Strumpfsysteme bestehen aus zwei Strümpfen. Der Unterziehstrumpf hat einen Ruhedruck von etwa 17–22 mmHg und sollte über Nacht getragen werden. Er fixiert den Wundverband und kann bei einigen Systemen auch als Anziehhilfe dienen (Abb. 4a). Darüber wird dann der eigentliche MKS angezogen (Abb. 4b). Dieser Überstrumpf entspricht meist einer KKL II, sodass sich der erwirkte Druck zur KKL III addiert [6]. Wenn das Ödem in der Entstauungsphase durch PKV reduziert wurde, ist ein stabiler Zustand erreicht. Es schließt sich dann die Erhaltungsphase an. Ulkus-Strumpfsysteme kommen nach Abschluss der Entstauungsphase für diese Erhaltungsphase bis zum vollständigen Wundverschluss zum Einsatz. Aus hygienischen Gründen sollte der Unterziehstrumpf täglich gewechselt werden [6]. Den Sets liegen daher 2 Unterziehstrümpfe bei. Viele Menschen sind nach einer kurzen Schulung in der Lage, diese Systeme selbstständig an- und abzulegen.

### Medizinische Kompressionsstrümpfe (MKS)

Wenn alle Wunden abgeheilt sind, beugt das Tragen von MKS insbesondere bei Menschen mit CVI der Rezidivprävention vor (Tab. 6). Generell nimmt der Druck bei MKS von distal nach proximal ab. Allerdings konnte für MKS am Unterschenkel mit ansteigendem Druckverlauf, d. h. einem höheren Druck im Waden- als im Knöchelbereich, ebenfalls eine Wirksamkeit bei CVI nachgewiesen werden [25]. MKS mit einem progressiven Druckverlauf haben deutlich stärkere Effekt auf die venöse Pumpfunktion als elastische MKS [12]. Meist wird der Einsatz von MKS der KKL II empfohlen, ggf. kann aber auch eine KKL I ausreichend sein [6, 21]. MKS bestehen aus Polyamid, Elastan, Baumwolle, Elastodien, Viskose oder Mikrofasern [22]. Sie sind im deutschsprachigen Raum in vier KKL nach RAL-Gütezeichen (Tab. 5; [23]) sowie in den Strickungen flach- oder rundgestrickt erhältlich. Entsprechend den aktuellen Leitlinienempfehlungen ist die jeweilige KKL entsprechend den klinischen Befunden und der Schwere der Beschwerden individuell festzulegen. Eine konkrete Zuordnung der Diagnosen zu einer KKL findet sich somit nicht mehr. Es wird vielmehr darauf hingewiesen, dass die niedrigste wirksame KKL rezeptiert werden sollte, da dies die Adhärenz gegenüber der Kompressionstherapie unterstützt. Je nach Indikation kommen MKS als konfektioniertes Fertigprodukt oder als Maßanfertigung zum Einsatz. Die sog. Konfektions‑, Serien- oder Normstrümpfe werden für das Bein in vier Ausführungen angeboten [22]:

- Wadenstrumpf (A–D),
- Halbschenkelstrumpf (A–F),
- Schenkelstrumpf (A–G),
- Strumpfhose (A–T).

**Table 6:** 

	Kurzzugbinden	Mehrkomponentensysteme	MAK	Ulkus-Strumpfsysteme	MKS
*Entstauung*	X	X	X	–	–
*Erhaltung*	–	–	X	X	–
*Prävention*	–	–	–	–	X

*MAK* medizinische adaptive Kompressionssysteme, *MKS* medizinische Kompressionsstrümpfe

Alle MKS haben eine definierte Länge (A bis D‑T). Für jeden Strumpftyp gibt es drei verschiedene Längen, um kurze, normale und lange Beine versorgen zu können. Bei Übereinstimmung der gemessenen Umfang- und Längenmaße mit den Normmaßen ist ein konfektionierter MKS auszuwählen. Relevant abweichende Maße bei Lymphödem erfordern häufig die Versorgung mit einem nach Maß angefertigten MKS, meist in Flachstrick [22].

Um das An- und Ausziehen, insbesondere von kräftigeren Flachstrickversorgungen in A–T-Ausführung zu erleichtern, ist die Verordnung von einzelnen Elementen, die nacheinander angezogen werden, beispielsweise Vorfußkappe, Capri‑/Radlerhose und Kniestrümpfen sowie die Einarbeitung von Reißverschlüssen empfehlenswert [22]. Die Vorteile der MKS entsprechen denen der 2‑lagigen Ulkus-Strumpfsysteme. Betroffene können aus einer Vielzahl an Farben, Musterungen oder Motivwebungen auswählen. Die Akzeptanz und Compliance bzw. Adhärenz der Betroffenen gegenüber der Kompressionstherapie wird durch die Möglichkeit, sich mit dem für den eigenen Geschmack passenden Produkt auszustatten, gefördert. Hierzu trägt auch die Verordnung einer An- und Ausziehhilfe bei [27]. Zudem gibt es auch spezielle MKS, die als sog. Angio-Strümpfe in KKL I mit hoher Stiffness erhältlich sind [28]. Weitere Modelle in KKL I und II sind zusätzlich in der Versorgung von Menschen mit Diabetes mellitus einsetzbar [26]. Zudem gibt es auch MKS, die speziell für Menschen mit ausschließlich Diabetes mellitus und Ödemen konzipiert sind. Diese liegen zwischen KKL I und II (18–25 mmHg) [29]. Diese speziellen MKS sind oft deutlich weicher, an der Fußsohle sowie im Zehenbereich gepolstert und nahtlos. Einige Hersteller bieten die MKS für Menschen mit Diabetes mellitus auch in hellen Farben wie beispielsweise weiß an, damit Betroffene insbesondere bei Polyneuropathie ggf. Verletzungen durch Verfärbungen im Strumpf erkennen können.

### Medizinische Thromboseprophylaxestrümpfe

Medizinische Thromboseprophylaxestrümpfe (MTPS) dienen der Prävention der Thromboembolie insbesondere bei immobilen Menschen und somit auch bei Bettruhe. Sie werden oft im perioperativen Bereich eingesetzt. Erhältlich sind MTPS als konfektionierte Produkte in verschiedenen Längen und Größen. Diese rundgestrickten nahtlosen Strümpfe bewirken im Liegen eine Reduzierung des Querschnitts der Venen, eine Beschleunigung des venösen Rückstroms und verbessern die Funktion der Venenklappen. Es handelt sich nicht um MKS. Dennoch bewirken sie einen effektiven Kompressionsdruck, der meist zwischen 13 und 18 mmHg liegt.

## An- und Ausziehhilfen

**Figure 5:**
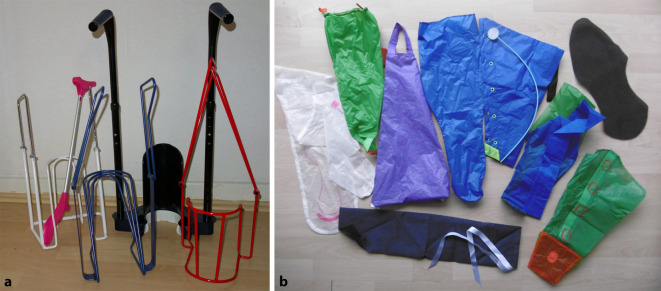

An- und Ausziehhilfen sind als Hilfsmittel bei entsprechender Indikation wie altersbedingter Kraftminderung grundsätzlich verordnungs- und erstattungsfähig [22]. Sie erleichtern den Betroffenen das An- und Ablegen der MKS und fördern so die Selbstpflegekompetenz. Zudem wird das Material geschont, und die Lebensdauer der MKS erhöht sich. Da es eine Vielzahl von An- und Ausziehhilfen gibt, sollte vorab eine individuelle Beratung erfolgen und ermittelt werden, welches Modell für die jeweiligen Bedürfnisse und körperlichen Fähigkeiten geeignet ist. Es gibt Systeme, die das An- und Ablegen von MKS mit offener und/oder geschlossener Spitze erleichtern, sowie solche, die lediglich das Anziehen oder das Ausziehen unterstützen. Generell können die aktuell verfügbaren Systeme in Gestelle (Abb. 5a) und Gleiter (Abb. 5b) unterschieden werden; zudem gibt es Sonderformen. Der zusätzliche Einsatz von genoppten Gummi- bzw. Haushaltshandschuhen ist eine weitere gute Unterstützung. Sie erhöhen die Griffigkeit und mindern das Risiko von Materialschäden beispielsweise durch Fingernägel.

## Intermittierende pneumatische Kompressionstherapie

**Figure 6:**
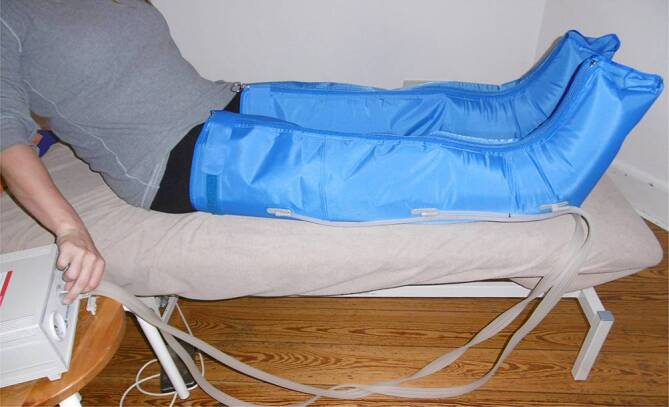

Die intermittierende pneumatische Kompressionstherapie (IPK) wird auch synonym als apparative intermittierende pneumatische Kompressionstherapie (AIK) bezeichnet. Die IPK wird beispielsweise als ergänzende Therapie bei Menschen mit UCV, in der Entstauungstherapie von Ödemerkrankungen sowie zur Thromboembolieprophylaxe empfohlen [24]. Es wird empfohlen, diese Methode unterstützend zu nutzen, wenn ein UCV mit fehlender Heilungstendenz trotz konsequenter Kompressionstherapie mittels Bestrumpfung oder PKV weiterhin besteht [24]. Bei der IPK füllt ein elektronisch gesteuertes System über eine Pumpe eine oder mehrere Luftkammern für einen kurzen Zeitraum auf (Abb. 6). Die so intermittierend befüllten Luftkammern befinden sich in einer Manschette, die temporär einen Druck auf ein bestimmtes Körperteil ausübt. Der über ein Steuergerät einstellbare und dokumentierbare Kompressionsdruck wird in definierten Zeitabständen von distal nach proximal sequenziell auf- und abgebaut. Durch abwechselndes Befüllen von mehreren, sich überlappenden Luftkammern entsteht so ein intermittierender, einstellbarer Behandlungsdruck, der die Wirkweise der Muskelpumpen simuliert und die Funktion der Venen und Lymphgefäße unterstützt sowie die arterielle Perfusion verbessert. Als neue Varianten gibt es Systeme, die ausschließlich für den Fuß oder den Oberschenkel konzipiert wurden.

## Fazit für die Praxis

- Für die Kompressionstherapie stehen heute viele verschiedene Materialien zur Verfügung.
- Bislang werden teils sehr unterschiedliche Begriffe genutzt, sodass eine einheitliche Nomenklatur sinnvoll erscheint.
- In diesem Expertenkonsens wird eine einheitliche Nomenklatur vorgeschlagen, die einen sachgerechten Austausch aller an der Versorgung von Menschen mit Kompressionstherapie Beteiligten gewährleisten soll.
- Bei der möglichst gemeinsam mit den Betroffenen getroffenen Entscheidung für bestimmte Materialien, sollten bestehende Symptome, individuelle Faktoren und wirtschaftliche Aspekte berücksichtigt werden.
